# Cross-cultural adaptation, reliability, and validity of the Turkish version of the obesity-specific quality of life questionnaire: quality of life, obesity, and dietetics (QOLOD) rating scale

**DOI:** 10.3906/sag-2005-412

**Published:** 2021-10-21

**Authors:** Nimetcan Mehmet YAĞMA, Egemen ÜNAL, Mehmet Enes GÖKLER, Salih MOLLAHALİLOĞLU

**Affiliations:** 1 Department of Public Health, Faculty of Medicine, Ankara Yıldırım Beyazıt University Turkey

**Keywords:** Obesity, QOLOD rating scale, QOL, Turkish version

## Abstract

**Background/aim:**

Obesity is one of the main public health issues in many countries including Turkey. The aim of the study is to test cross-cultural adaptation, reliability, and validity of QOLOD rating scale in the Turkish language.

**Materials and methods:**

This methodological study was conducted among the overweight and obesity people between February-March, 2018 in Ankara Atatürk Training and Research Hospital. The data was collected through self-report and face to face interviews. The QOLOD rating scale has 36 items, a 5-point Likert scale (1–5) is used for each question.

**Results:**

In the study, of the 180 participants, 101 (56.1%) were female, 79 (43.9%) were male, and the mean age was 43.36 ± 14.28 (min-max 18–87) years. According to the CFA, the Turkish version of QOLOD rating scale shows a multidimensional structure consisting of 34 items. Two items (item 11 and item 35) were excluded from the scale according to the CFA. Cronbach’s Alpha value changes between 0.927–0.930.

**Conclusion:**

Finding shows that the Turkish version of QOLOD rating scale had sufficient validity and reliability for Turkish population, had strong psychometric characteristics.

## 1. Introduction 

Overweight and obesity is one of the main public health issues in many countries [1]. Obesity is a serious health issue of 21st century and is an important cause of morbidity and mortality in all age group [2]. According to World Health Organization (WHO), worldwide obesity has nearly tripled since 1975 and in 2016, more than 1.9 billion adults, 18 years and older, were overweight. Of these over 650 million were obese. Most of the world’s population live in countries where overweight and obesity kills more people than underweight World Health Organization (2019). Facts and figures on childhood obesity: Commission on Ending Childhood Obesity. [online] Website https://www.who.int/news-room/fact-sheets/detail/obesity-and-overweight [accessed April, 2020].. Body mass index (BMI), defined as body mass in kg divided by the square of height in meters (kg/m^2^), is the most commonly used anthropometric measure to approximate overall body fatness for the purposes of classifying and reporting overweight and obesity [3]. BMI is a statistical index using a person’s weight and height to provide an estimate of body fat in males and females of any age. BMI classified as severely underweight - BMI less than 16.5 kg/m^2^, underweight - BMI under 18.5 kg/m^2^, normal weight - BMI greater than or equal to 18.5 to 24.9 kg/m^2^, overweight – BMI greater than or equal to 25 to 29.9 kg/m^2^, obesity – BMI, greater than or equal to 30 kg/m^2^ and obesity [4]. Obesity is one of the most important reasons for reduced life expectancy within the “modern” world. The prevalence of overweight and obesity continues to increase both in developing and in developed countries [5]. Weight gain usually progressively increases in adults from about the age of 20 years, and it is now clear that on a global level the prevalence in men peaks at about 50–55 years and in women at about 60 years [6]. Obesity and overweight, as a part of the metabolic syndrome, are well known risk factors for the development of diabetes, hypertension, coronary heart disease, hyperlipidemia, stroke, sleep apnea syndrome, osteoarthritis, and certain forms of cancer [7]. Obesity usually results from a combination of causes and contributing factors such as, lifestyle choices, unhealthy diet, positive energy balance, inactivity, certain disease and medications and social and economic issues [8]. Turkey also facing challenges like other countries, the prevalence of overweight and obesity in adults and children has substantially increased over the past two decades in the country. Overweigh among adults is more common among men and obesity is more prevalent among women in Turkey. Among children, the prevalence of obesity was similar in both sexes [9]. Overweightness and obesity effect to quality of life in general. Overweightness and obesity have the largest association with physical function measures [10]. Obesity is associated with worse health related to quality of life (HRQOL), especially in women and people aged over 64 years [11]. A new questionnaire, called the “quality of life, obesity, and dietetics (QOLOD)” rating scale was developed [12]. It is important to Cross-cultural adaptation, reliability, and validity of the Turkish version of the obesity-specific quality of life questionnaire since obesity is one of main public health issue in the country. The Turkish version could be useful for further studies in the relevant studies. The aim of the study is to test cross-cultural adaptation, reliability, and validity of QOLOD rating scale in Turkish Language. 

## 2. Materials and methods

### 2.1. Study population and location

The cross-cultural adaption study was carried out among the overweight and obesity people between February–March, 2018 in Ankara Ataturk Training and Research Hospital. Trained researchers observed the people if there were any overweigh and obesity who were waiting for their health services in different department and unite in the hospital. After the researchers identified overweight and obesity people then they explained the study briefly to them. If they agreed to participate to the study, then the questioner was given to them or face to face interview was done with them. The data was collected through self-report and face to face interview. The researchers waited until the participant filled up the form. The study sample was 180 individuals based on the recommendation that the number of samples be 5–10 times greater than that of scale items [13]. Overweight and obesity people, aged 18 years and above, and who were agreed to participate to the study were included. Their weight and height were based on participant’s report. We accepted the weight in kg and height in cm. The presence of chronic disease of the participants was evaluated according to the statements of the participants. Those with at least one chronic disease were rated as having “chronic disease”.

### 2.2. The QOLOD rating scale

The QOLOD rating scale has 36 items, out of 11 questions related to Physical impact, 11 questions related to the psychosocial impact, 4 questions related to impact of sex life, 5 questions related to comfort with food, and 5 questions related to diet experience. A 5-point Likert scale (1–5) is used for each question, it was graded from 1 to 5 (1: always/enormously; 2: often/a lot; 3: sometimes/moderately; 4: rarely/a little; 5: never/not at all). A score was then calculated for each dimension by adding together its constituent items. Scores obtained by adding up answers graded from 1 to 5 of all items per dimension were transformed to convert the lowest and highest. The higher the score represents the better the quality of life.

The QOLOD rating scale was translated from English language to Turkish language according to the standard methodology recommended by researchers [14]. It was translated by health professions who are fluent in both languages. It was translated from English to Turkish and then it was translated back to English to compare the both version of the questionnaire. 

### 2.3. Statistical analysis

Data were analyzed using the IBM Statistical Package for Social Sciences (SPSS), v. 24.0 and Lisrel for Windows (student bersion). Mean and standard deviation were reported for numerical variables and descriptive statistics frequency and percentage were reported. 

For validity analysis of the Turkish version of QOLOD rating scale, Kaiser–Meyer–Olkin (KMO) value was determined and Bartlett’s test was used. Confirmatory factor analysis (CFA) was used for construct validity. Confirmatory factor analysis (CFA) was undertaken to evaluate how well the model fits into the observed data, that is, whether the proposed model fits the data. The practical indicators of fit according to degrees of freedom (χ²/df), goodness of fit index (GFI), mean square error of approximation residual (RMSEA), root mean square residual (RMR), standardized root mean square residual (SRMR), normed fit index (NFI), and comparative fit index (CFI). Kruskal–Wallis and Mann–Whitney U analysis was used to assess the means of the groups for clinical validity analysis.

Item total score correlation was used to evaluate the reliability of the scale. For the reliability analysis and internal consistency of the Turkish version of QOLOD rating scale, Cronbach’s Alpha test was used. The results were evaluated within the 95% confidence interval and p ≤ 0.05 were considered as statistically significant.

### 2.4. Ethics clearance

Permission was obtained from the authors of original paper through email and the study proposal was approved by the Ethical Committee of Ankara Yildirim Beyazit University. All necessary permissions were obtained from Ankara Ataturk Training and Research Hospital. Informed and written consent was obtained from all participants.

## 3. Results

In the study, of the 180 participants, 101 (56.1%) were female, 79 (43.9%) were male, and the mean age was 43.36 ± 14.28 (min-max 18–87) years. The average score of the individuals on the QOLOD rating scale was 112.78 ± 24.88 (min-max 46–175). While the scores obtained from the Turkish version of QOLOD rating scale showed no correlation with the ages of the participants (r = 0.131; p = 0.079), the scores were found higher in men than in women (MWU=2.860; p < 0.001). The majority of the individuals participating in the study were obese. The BMI score average of the study group was 33.65 ± 4.98 (min-max 25.26–60.19). The scores obtained from the QOLOD rating scale showed no correlation with the BMI scores of the individuals (r =- 0.121; p = 0.106). 

### 3.1. Validity analysis of the Turkish version of QOLOD scale 

In the study, Kaiser–Meyer–Olkin (KMO) value was determined as 0.883 (p < 0.001). Bartlett’s test of sphericity had a result of 4,351,350 with p < 0.001. With these results, the scale data was found to be compatible for CFA. According to the CFA, the Turkish version of QOLOD rating scale shows a multidimensional (5 dimensions as physical impact, psycho-social impact, impact on sex life, comfort with food, diet experience) structure consisting of 34 items. Two items (item 11 and item 35) were excluded from the scale according to the CFA. 

A CFA was conducted in order to test QOLOD rating scale’s 5-factor model. Several test statistics were used in the CFA to determine the adequacy of model to fit data such as degrees of freedom (χ²/df), goodness of fit index (GFI), Mean square error of approximation residual (RMSEA), root mean square residual (RMR), standardized root mean square residual (SRMR), normed fit index (NFI), incremental fit index (IFI), and comparative fit index (CFI). In addition, modifications were made with second level CFA analysis in line with modification suggestions. While making improvements, variables that decrease compliance were determined, and new covariances were created for those with high covariance. Poor fit was observed based on the GFI (0.74), but moderate/good fit occurred with other indices: X^2^/df (1068.84/517 = 2.07), RMSEA (0.077), RMR (0.12), SRMR (0.074), NFI (0.90), CFI (0.94), IFI (0.95). Goodness of fit statistics indicates that QOLOD rating scale is acceptable.

### 3.2. Clinical validity of the scale

In order to test the clinical validity of the scale, hypothetical comparisons of the sociodemographic characteristics and some clinical features of the study group was made. In our study, no statistical difference was observed between the scores obtained by obese and overweight individuals from the QOLOD rating scale (p = 0.517).

The individuals participating in the study were evaluated in two age groups (under 60 age & 60 age and above). According to this analysis, no statistical difference was observed between the scores of participants under age 60 and age 60 and above from the QOLOD rating scale (p = 0.561). Additionally, in our study, the QOLOD rating scale scores of participants who have higher educational status were evaluated as statistically similar with participants who have lower educational status (p = 0.333). In the study, the QOLOD rating scale scores of the participants with high income status were higher than the participants with middle and low income levels (p < 0.001). However, the scores of middle and low income individuals were evaluated as similar (p > 0.05). It was determined that the participants who had children had higher QOLOD rating scale scores than the participants who did not have children (p = 0.004). However, it was found that the number of children did not correlate with the scores obtained from the scale (p = 0.132). In this current study there was no difference between the groups in terms of scores obtained from the scale according to the status of having chronic disease (p = 0.100). Table 1 shows distribution of some clinical and sociodemographic characteristics of the study group according to the scores obtained from the QOLOD rating scale.

**Table 1:** Distribution of some clinical and sociodemographic characteristics of the study group according to the scores obtained from the QOLOD rating scale.

		N (%)	Mean ± SD	Median	Min.-Max	Test valueKW/Z;p
BMI status	Obese	138 (76.7)	111.91 ± 24.55	110.0	46.0–172.0	2.706; p = 0.517
	Overweight	42 (23.3)	115.64 ± 26.04	115.5	74.0–172.0	
Age group	Under 60 age	158 (87.8)	112.25 ± 24.88	111.5	46.0–175.0	1.871; p = 0.561
	60 age and above	22 (12.2)	116.59 ± 25.11	107.5	79.0–172.0	
Education status	Secondary school and below	47 (26.1)	109.93 ± 23.37	106.0	67.0–171.0	3.421; p = 0.335
	High school and above	133 (73.9)	113.78 ± 25.40	112.0	46.0–175.0	
Income status	Low	62 (34.4)	105.30 ± 21.24	103.5	73.0–171.0	15.673; p < 0.001
	Medium	87 (48.3)	113.27 ± 25.83	111.0	46.0–175.0	
	High	31 (17.2)	126.35 ± 23.60	131.0	80.0–172.0	
Having a child	No	57 (31.7)	104.80 ± 21.62	104.0	62.0–156.0	2.573; p = 0.004
	Yes	123 (68.3)	116.48 ± 25.50	113.0	46.0–175.0	
Having a chronic disease	No	96 (53.3)	115.49 ± 26.94	115.0	46.0–175.0	4.605; p = 0.100
	Yes	84 (46.7)	109.69 ± 22.05	107.0	70.0–172.0	

### 3.3. CFA and reliability analysis of the Turkish version of QOLOD rating scale

In our final CFA of the 34 items limited to 5 factors confirmed that items were in general well distributed in the original study dimensions as “physical impact, psychological impact, sexual impact, comfort with food, diet experience”. All Cronbach’s alpha coefficients of sub-dimensions of the scale were above 0.8, so confirming the good internal reliability of the scale. Figure shows the results of confirmatory factor analysis of the 5–factor model of the QOLOD rating scale and also Turkish form of the QOLOD scale is shown in Table 2.

**Table 2:** Turkish form of the QOLOD scale (Obezite Hastalarına Yönelik Yaşam Kalitesi Ölçeği).

No.	Madde	Her zaman(1)	Sık sık(2)	Bazen (3)	Nadiren(4)	Asla(5)
	Fiziksel etki (Q1-Q11)Kilom yüzünden ...					
Q1	Nefes almakta zorluk çekiyorum					
Q2	Günün sonunda ayak bileklerim ve bacaklarım şişiyor					
Q3	Fiziksel aktivite gösterdiğimde göğüsüm ağrıyor					
Q4	Eklemlerimde sertlik ve ağrı ile ilgili sorunum var					
Q5	Görevlerimi yapmakta zorluk çekiyorum ya da sorumluluklarımı yerine getirmekte zorlanmaktayım					
Q6	Bedensel sağlık durumumu zayıftır					
Q7	Sırtım ağrıyor					
Q8	Soyunma ve giyinme konusunda zorluk çekiyorum					
Q9	Ayakkabı bağı bağlamada zorluk çekiyorum.					
Q10	Merdivenden çıkarken zorlanıyorum					
Q11	Olabileceğimden daha az hareketliyim (item excluded from the scale in analysis phase)					
	Psiko-sosyal etki (Q12-Q22)Kilom yüzünden					
Q12	Yalnız zaman geçiriyorum ya da içime kapalıyım					
Q13	İş görüşmelerine gitmekten çekiniyorum					
Q14	Kendimi eğlendirmekte zorlanıyorum					
Q15	Kendimi depresyonda hissediyorum, moralim iyi değil					
Q16	İrade eksikliğim var					
Q17	İnsanlar benim için “çok hoş” biri ama ‘çok zeki biri değil’ diye düşünüyor					
Q18	Kilomdan utanıyorum					
Q19	Başkalarına göre kendimi daha aşağıda hissediyorum					
Q20	İnsanlar benim yemek yediğimi gördüğünde kendimi suçlu hissediyorum.					
Q21	Giysisiz görünmek istemiyorum					
Q22	Bana uygun bedende ve yakışan kıyafetler bulmakta zorlanıyorum					
	Cinsel yaşam üzerindeki etkiler (S223-Q26)Kilom yüzünden ...					
Q23	Cinsel isteğim az ya da hiç yok					
Q24	Cinsel ilişki sırasında fiziksel zorluk yaşıyorum					
Q25	Mümkün olduğunca cinsel ilişkiden kaçınıyorum					
Q26	Cinsel ilişkiden zevk almıyorum					
	Yemekle rahat olma (Q27-Q31)					
Q27	Yemeyi severim					
Q28	Yemekten sonra memnuniyet hissediyorum					
Q29	Yemek beni iyi hissettirir ve bana zevk verir					
Q30	Yemek yeme fikrinden memnun olurum					
Q31	Yemek yeme düşüncesini severim					
	Diyet tecrübesi (Q32-36)					
Q32	Diyet benim için yoksunluk ve hayal kırıklığı demektir					
Q33	Diyet beni ailemle ve / veya arkadaşlarımla birlikte istediğim yemeği yememi engelliyor					
Q34	Diyet beni yoruyor ve sağlıksız görünmeme sebep oluyor					
Q35	Yememem gereken yiyecekleri ne zaman yesem suçlu hissediyorum(item excluded from the scale in analysis phase)					
Q36	Diyet beni agresif/sinirli yapıyor					

Note: A five-point Likert scale (1–5) is used for each question, it was graded from 1 to 5 (1: always/enormously; 2: often/a lot; 3: sometimes/moderately; 4: rarely/a little; 5: never/not at all). A score was then calculated for each dimension by adding together its constituent items. Scores obtained by adding up answers graded from 1 to 5 of all items per dimension were transformed to convert the lowest and highest (36–180). The higher the score represents the better the quality of life. The total score can be evaluated between 0 and 100 percentages. In our revised scale, we excluded two items from the scale (11 and 35). So the total score ranges between 34 and 170. Researchers can also use the excluded items in their research. Our recommendation is to use it by removing two items.

**Figure 1:**
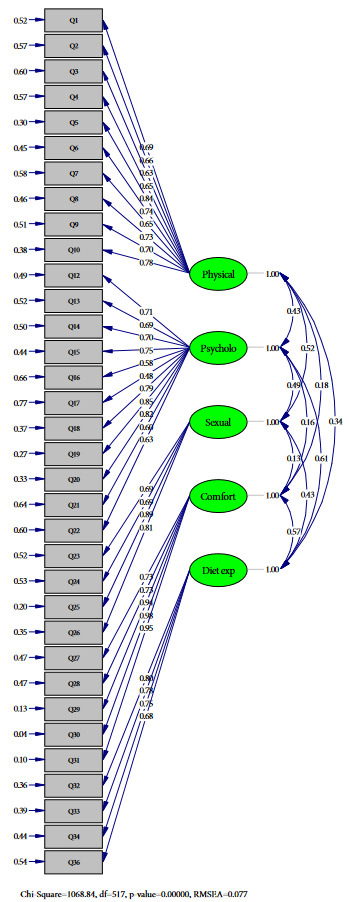
The results of confirmatory factor analysis of the five–factor model of the QOLOD rating scale.

## 4. Discussion

This methodological study aimed to assess the reliability and validity of the Turkish version of QOLOD rating scale developed by Ziegler et al. on the French population [12]. The first psychometric results show that the Turkish version of QOLOD rating scale has sufficient characteristics in terms of its validity and reliability. According to the CFA, poor fit was observed based on the GFI (0.74), but moderate/good fit occurred with other indices: X2/df (1068.84/517=2,07), RMSEA (0.077), RMR (0.12), SRMR (0.074), NFI (0.90), CFI (0.94), IFI (0.95) [15]. Goodness of fit indices are usually a measure of the amount of variance and covariance explained by the model. It can be said that the closer the value of the goodness of fit indexes to 1, the more the model is compatible with the data. 0.90–0.95 acceptable for goodness of fit indices and being over 0.95 indicates a high fit [16,17]. On the other hand, the other model’s indices can be accepted between 0.08–0.05 values; It can be said that the model is good even when they are less than 0.05. Especially the RMSEA index value close to 0.00 is a good fit show [16,17]. Goodness of fit statistics indicates that is QOLOD rating scale is acceptable.

Similar to the original study in the Turkish version of the scale, we evaluated 5 dimensions that express the quality of life and diet-related experiences of obese and overweight people in their daily lives. In our study 

We excluded the 11th and 35th items which belonging to the “physical impact and diet experience” dimensions from the Turkish version of the scale because of their load factor below 0.4. The first item removed from the scale is item 11 (“I am less effective than I could be”). In the evaluations made during the translation phase, no problem was detected for the Turkish version of the item. However, in the analysis results, the item was excluded from the Turkish version of the scale because the question disrupted the modeling of the scale and the item was not fully understood by the participants. According to our assessment, the Turkish form of the item is not very clear. The other removed item is item 35 (I feel guilty whenever I eat foods that I should not). When the analysis results were examined, while the consistency between the items was noticeable in this sub-dimension of the scale, the factor load of 35th item was low. According to our assessment the concept of guilt in this item was perceived as disturbing by the participants and the responses were different from other sub-dimension items. The item was evaluated separately from other items in the “diet experience” sub-dimension by the participants. As a matter of fact, the load of this factor was below the other items in the original scale study (0.44) [12].

In validity analysis to test the clinical validity of the scale, no statistical difference was observed between the scores obtained by obese and overweight individuals from the QOLOD rating scale. This result may be due to obese and overweight people performing similar daily activities and having similar health problems and difficulties in these daily activities. Many studies show that overweight individuals will become obese in the future [18–20]**.** The scale is not expected to diagnose the obese and overweight group. In the study we evaluated the overweight group as people with similar sociocultural and psychological background in terms of susceptibility to obesity, so we formed the study group from these two groups.

In our study, no statistical difference was observed between the scores of participants under aged 60 and above 60 from the Turkish version of QOLOD rating scale. In our opinion, hypothetically, overweight and obese individuals who over a certain age are expected to have more physical function losses and have lower quality of life due to obesity and effect of additional chronic diseases [10]. However, it is reported that the high mass, especially due to obesity, protects the bone mass, and prevents the expected physical function loss and muscle loss at a later age [21]**.** The scale scores of male participants are higher than women support this hypothesis. In addition, no difference was observed between the scores of participants with or without chronic disease. This result shows that obesity affects people’s quality of life regardless of age group and chronic diseases status. Additionally, in our study, the QOLOD rating scale scores of participants who have higher educational status were evaluated as statistically similar with participants who have lower educational status. We believe that the main difference should be the opportunities and resources that individuals have in dealing with the difficulties created by obesity. To explain this, in the study, the scores of the participants who evaluated the income status as high were higher than the individuals with middle and low income levels. This result shows that individuals with high income are more advantageous in dealing with the effects of the disease on the quality of life. Individuals with high income can access effective treatment opportunities, activities that can positively affect quality of life, and tools and equipment that make their daily life easier. According to the status of having children, it was determined that the participants who had children had higher scores on the Turkish version of QOLOD rating scale. Being a family with children can provide opportunities and healthy environments to tolerate loss of quality of life caused by obesity and overweight [22]**.** According to the reliability analysis of the Turkish version of QOLOD rating scale, Cronbach’s alpha coefficient of the Turkish version of QOLOD rating scale consisting of 34 items was determined to be 0.930. Cronbach alpha coefficient was evaluated as very sufficient [23]. In addition, all the sub-dimensions (physical impact, psychological impact, sexual impact, comfort with food, diet experience) of the scale had Cronbach alpha coefficient above 0.8. These coefficients were found higher than the original scale study [12]. The Turkish version of QOLOD rating scale was found valid and reliable for Turkish-speaking overweight and obesity patients in the present study. Could be information bias may occur since participant’s weight and height were based on their report so this could be a limitation of the study.

In conclusion, in our study showed that the Turkish version of QOLOD rating scale had sufficient validity and reliability for Turkish population, had strong psychometric characteristics. The results suggested that the Turkish version QOLOD rating scale is a good psychometric tool to assess and improve individuals’ obesity-related quality of life. The scale has been adapted to the sociocultural factors of obesity and overweight in the Turkish population and its validity and reliability have been tested. This scale helps clinicians to see the effects of dietary management on the quality of life of obese people.

## Informed consent

Informed consent was prepared for the participants. The prepared form was verbally expressed to the participants clearly. Verbal consent was obtained from them.
